# Supplementary material for: High donor hemoglobin interacts with pre-transplant recipient neutropenia to modulate mortality after allogeneic hematopoietic stem cell transplantation: An exploratory, single-center, retrospective, real-world study
Source: PLoS One. 2026 May 22;21(5):e0349615. doi: 10.1371/journal.pone.0349615 (PMC13196987; doi:10.1371/journal.pone.0349615)
Supplement: S1 Table — (DOCX) [file pone.0349615.s001.docx]

**Table S1: sensitivity analysis comparing multiple imputation (MI) and complete case analysis (CCA) results for uric acid and ALP (adjusted for age and aender)**

| **Factor** | **Analysis Method** | **N** | **Adjusted Hazard Ratio (aHR)** | **90% Confidence Interval** | **P-value** |
| --- | --- | --- | --- | --- | --- |
| **Uric Acid (continuous)** |  |  |  |  |  |
|  | CCA | 77 | 0.759 | 0.593 to 0.972 | 0.066 |
|  | MI | 94 | 0.768 | 0.605 to 0.975 | 0.070 |
| **Alkaline Phosphatase (continuous)** |  |  |  |  |  |
|  | CCA | 81 | 1.002 | 0.9996 to 1.004 | 0.168 |
|  | MI | 94 | 1.002 | 0.9997 to 1.004 | 0.146 |

*Abbreviations: MI, Multiple Imputation; CCA, Complete Case Analysis; aHR, Adjusted Hazard Ratio; CI, Confidence Interval; ALP, Alkaline Phosphatase.* *Note: All models adjusted for recipient age and gender.*
