# Supplementary material for: High donor hemoglobin interacts with pre-transplant recipient neutropenia to modulate mortality after allogeneic hematopoietic stem cell transplantation: An exploratory, single-center, retrospective, real-world study
Source: PLoS One. 2026 May 22;21(5):e0349615. doi: 10.1371/journal.pone.0349615 (PMC13196987; doi:10.1371/journal.pone.0349615)
Supplement: S1 Checklist — (DOCX) [file pone.0349615.s002.docx]

**STROBE Statement—Checklist of Items for a Cohort Study**

**Manuscript Title:** High donor hemoglobin interacts with pre-transplant recipient neutropenia to modulate mortality after allogeneic hematopoietic stem cell transplantation: an exploratory, single-center, retrospective, real-world study

| **Item No** | **Recommendation** | **Location and Description** |
| --- | --- | --- |
| **Title and abstract** |  |  |
| 1a | **Indicate the study’s design with a commonly used term in the title or the abstract** | **Title and Abstract.** We explicitly state the study design in the title as a "single-center, retrospective, real-world study". The abstract further clarifies the design as a "retrospective cohort study" (Page 2, Abstract). |
| 1b | **Provide in the abstract an informative and balanced summary of what was done and what was found** | **Abstract (Page 2).** The abstract provides a structured summary detailing the study's objective, methods (design, participants), key quantitative findings including the paradoxical main effect of donor hemoglobin (aHR 1.45, p=0.148) and the central interaction term (aHR 0.44, p=0.013), and a balanced conclusion on the implications and the crucial need for validation. |
| **Introduction** |  |  |
| 2 | **Explain the scientific background and rationale for the investigation being reported** | **Introduction (Page 3).** We detail the clinical context of allo-HSCT, summarize established prognostic factors (e.g., NLR), identify the critical knowledge gap concerning the interplay between host vulnerability and donor graft characteristics (the "soil and seed" concept), and thereby establish the scientific rationale for our investigation. |
| 3 | **State specific objectives, including any prespecified hypotheses** | **Introduction (Page 3, final paragraph).** We state a clear primary objective: to investigate the pre-specified hypothesis of an interaction between pre-transplant recipient neutropenia and donor hemoglobin levels. We also state our secondary objective: to identify other independent predictors of mortality in our cohort. |
| **Methods** |  |  |
| 4 | **Present key elements of study design early in the paper** | **Methods (Page 3, 'Study Design and Participants').** The study design is presented at the beginning of the Methods section as a "single-center, retrospective cohort study" conducted in accordance with the STROBE guidelines. |
| 5 | **Describe the setting, locations, and relevant dates, including periods of recruitment, exposure, follow-up, and data collection** | **Methods (Pages 3-4).** We describe the setting as the Bone Marrow Transplant Center of Imam Reza Hospital in Kermanshah, Iran. We specify the patient recruitment period as "between September 5, 2015 and March 12, 2024" and the final date of follow-up as "February 28, 2025". |
| 6 | **Participants: (a) Give the eligibility criteria, and the sources and methods of selection of participants.** | **Methods & Results (Pages 3 & 6, Figure 1).** Eligibility criteria are detailed, starting with all consecutive patients (n=116) undergoing allo-HSCT for hematological malignancies. Exclusion criteria (incomplete records >30% or unavailable survival information) are explicitly stated. This process is fully visualized in the STROBE flow diagram (Figure 1), as referenced on Page 6. |
| 7 | **Variables: Clearly define all outcomes, exposures, predictors, potential confounders, and effect modifiers.** | **Methods (Page 4, 'Data Collection and Definitions').** All key variables are clearly defined. The primary outcome (5-year overall survival) is defined. The primary exposure variable (neutropenia, as absolute neutrophil count <1,500/μL) and other predictors are specified. Potential confounders adjusted for *a priori* (recipient age and sex) are also explicitly stated. |
| 8 | **Data sources/measurement** | **Methods (Page 4, 'Data Collection, Variables, and Outcomes').** We specify that data were manually extracted from institutional health records by two trained assistants. To ensure measurement consistency and quality, we report a high inter-rater reliability (Cohen's Kappa κ = 0.92). |
| 9 | **Bias: Describe any efforts to address potential sources of bias** | **Methods (Pages 4-5).** We addressed potential biases in several ways. To control for confounding, key demographic variables (age, sex) were included in all multivariable models. To mitigate bias from missing data, we employed multiple imputation by chained equations (MICE). To prevent overfitting in our modest sample size, we adhered to the events-per-variable (EPV) guideline. The limitations section (Pages 12-13) discusses biases inherent to the design (e.g., unmeasured confounding) and unmeasured confounding. |
| 10 | **Study size: Explain how the study size was arrived at** | **Methods (Page 5).** Our study size was determined by including all consecutive eligible patients over a defined 8.5-year period. While an *a priori* sample size calculation was not applicable for this retrospective design, a *post-hoc* power calculation was performed, indicating that our study had 81.4% power to detect the observed unadjusted difference in the primary outcome. |
| 11 | **Quantitative variables: Explain how quantitative variables were handled in the analyses.** | **Methods (Page 4) & Results (Page 6).** We detail our handling of quantitative variables. Key variables like donor hemoglobin and liver enzymes were analyzed as continuous variables in the regression models (e.g., hazard ratio per 1 g/dL increase). For specific log-rank tests and for visualization purposes (Figure 4), we described the rationale for categorizing variables based on established or data-driven cutoffs (e.g., cohort median). |
| 12 | **Statistical methods: (a) Describe all statistical methods, including those used to control for confounding (b) Describe any methods used to examine subgroups and interactions (c) Explain how missing data were addressed (e) Describe any sensitivity analyses** | **Methods (Pages 4-5, 'Statistical Analysis') & Page 8 ('Model Robustness').** We provide a comprehensive description of our statistical approach. **(a)** Gompertz parametric models were used for multivariable analysis, adjusting for *a priori* confounders. **(b)** Our primary analytical goal was to test a pre-specified interaction term. We also report exploratory subgroup analyses. **(c)** Missing data were handled using MICE. **(e)** We conducted several sensitivity analyses, including a complete-case analysis, sequential addition of transplant covariates, and a replication of our interaction finding using a semi-parametric Cox model to ensure robustness. |
| **Results** |  |  |
| 13 | **Participants: (a) Report numbers of individuals at each stage of study (c) Consider use of a flow diagram** | **Results (Page 6, 'Cohort Derivation...').** We report the flow of participants, from the initial 116 assessed for eligibility to the final cohort of 94 included in the analysis, stating reasons for exclusions at each stage. **(c)** This information is visually presented in a STROBE flow diagram (Figure 1). |
| 14 | **Descriptive data: (a) Give characteristics of study participants and information on exposures and potential confounders. (b) Indicate number of participants with missing data for each variable of interest. (c) Summarise follow-up time** | **Results (Page 6) & Table 1 (Page 20-21).** **(a)** Baseline demographic, clinical, transplant, and laboratory characteristics are fully detailed in Table 1. **(b)** Table 1 and the Methods section explicitly state the number of patients with available data for variables where missingness occurred (e.g., N=92 for BMI). **(c)** We report a median follow-up of 33.6 months (IQR, 4.7–61.7). |
| 15 | **Outcome data: Report numbers of outcome events or summary measures over time** | **Results (Page 6).** We report a total of 30 mortality events (31.9% of the cohort) occurring during the follow-up period. The estimated 1-year, 3-year, and 5-year overall survival rates (71.3%, 67.3%, and 67.3%, respectively) are explicitly provided. |
| 16 | **Main results: (a) Give unadjusted estimates and, if applicable, confounder-adjusted estimates and their precision. (b) Report category boundaries when continuous variables were categorized** | **Results (Pages 6-8), Table 2 (Page 22-23), Figure 3.** **(a)** We present initial univariate analyses (log-rank p-values and unadjusted Gompertz HRs) in Table 2. Our primary, confounder-adjusted results (aHRs with 95% CIs and p-values) are presented in the text and visualized in Figure 3. The main effect of donor hemoglobin and the crucial interaction term are both reported with their precise statistical values. **(b)** Category boundaries are defined for all dichotomized variables (e.g., Uric Acid >7.3 mg/dL). |
| 17 | **Other analyses: Report other analyses done—e.g., analyses of subgroups and interactions, and sensitivity analyses** | **Results & Discussion (Pages 8-9), Figure 5.** We report the pre-specified interaction analysis as a central finding. We also present further exploratory subgroup analyses (e.g., effect of neutropenia stratified by age) in Figure 5. The results of our key sensitivity analyses (Cox model, complete-case analysis) are reported on Page 8 to confirm the robustness of our findings. |
| **Discussion** |  |  |
| 18 | **Key results: Summarise key results with reference to study objectives** | **Discussion (Page 9, first paragraph).** The Discussion section opens with a concise summary of our principal findings in direct reference to our objectives: the identification of higher donor hemoglobin as a paradoxical independent risk factor for mortality, and the significant qualitative interaction with recipient neutropenia that reverses this effect. |
| 19 | **Limitations: Discuss limitations of the study, taking into account sources of potential bias or imprecision. Discuss both direction and magnitude of any potential bias** | **Discussion (Page 12-13, 'Limitations').** We dedicate a substantial section to discussing the study's limitations, including its modest sample size, the retrospective, single-center design (limiting generalizability and exposing it to unmeasured confounding), lack of granular data on HCT-CI/ECOG, and the lack of granular data to directly test our biological hypotheses. |
| 20 | **Interpretation: Give a cautious overall interpretation of results considering objectives, limitations, multiplicity of analyses, results from similar studies, and other relevant evidence** | **Discussion (Pages 9-12).** We provide a cautious interpretation, contextualizing our findings within current biological understanding (e.g., EPO signaling). We explicitly state that our findings are hypothesis-generating and "should not, under any circumstances, be used to guide current clinical practice" (Page 12), thereby balancing our novel findings with their current limitations. |
| 21 | **Generalisability: Discuss the generalisability (external validity) of the study results** | **Discussion (Page 12).** We directly address generalizability as a key limitation, stating that "Findings from our unique regional cohort in Western Iran has a high prevalence of red blood cell disorders (like thalassemia minor) and heavily pre-treated patients.” We call for validation in larger, multi-center databases to establish external validity. |
| **Other information** |  |  |
| 22 | **Funding: Give the source of funding and the role of the funders for the present study** | **Funding (Page 14).** We provide a dedicated "Funding" section that explicitly states, "This research received no specific grant from any funding agency in the public, commercial, or not-for-profit sectors." |
